# Regulation of Bimetallic Coordination Centers in MOF Catalyst for Electrochemical CO_2_ Reduction to Formate

**DOI:** 10.3390/ijms241813838

**Published:** 2023-09-08

**Authors:** Rui Yang, Qun Huang, Xuelan Sha, Beibei Gao, Juan Peng

**Affiliations:** State Key Laboratory of High-Efficiency Utilization of Coal and Green Chemical Engineering, College of Chemistry and Chemical Engineering, Ningxia University, Yinchuan 750021, China

**Keywords:** electrocatalysis, carbon dioxide, formate, coordination centers, bimetallic

## Abstract

Electrocatalytic reduction of CO_2_ to valuable chemicals can alleviate the energy crisis, and solve the greenhouse effect. The key is to develop non-noble metal electrocatalysts with high activity, selectivity, and stability. Herein, bimetallic metal organic frameworks (MOFs) materials (BiZn-MOF, BiSn-MOF, and BiIn-MOF) were constructed by coordinating the metals Zn, In, Sn, and Bi with the organic ligand 3-amino-1H-1,2,4-triazole-5-carboxylic acid (H_2_atzc) through a rapid microwave synthesis approach. The coordination centers in bimetallic MOF catalyst were regulated to optimize the catalytic performance for electrochemical CO_2_ reduction reaction (CO_2_RR). The optimized catalyst BiZn-MOF exhibited higher catalytic activity than those of Bi-MOF, BiSn-MOF, and BiIn-MOF. BiZn-MOF exhibited a higher selectivity for formate production with a Faradic efficiency (FE = 92%) at a potential of −0.9 V (vs. RHE, reversible hydrogen electrode) with a current density of 13 mA cm^−2^. The current density maintained continuous electrolysis for 13 h. The electrochemical conversion of CO_2_ to formate mainly follows the *OCHO pathway. The good catalytic performance of BiZn-MOF may be attributed to the Bi-Zn bimetallic coordination centers in the MOF, which can reduce the binding energies of the reaction intermediates by tuning the electronic structure and atomic arrangement. This study provides a feasible strategy for performance optimization of bismuth-based catalysts.

## 1. Introduction

The excessive emission of CO_2_ is the main cause of the greenhouse effect, which poses a serious threat to global ecology. Converting excessive CO_2_ emissions into valuable chemicals and fuels is an effective means of solving both environmental and energy crises [1,2]. One of the most attractive methods is the electrochemical CO_2_ reduction reaction, which uses renewable electricity to convert CO_2_ into commercially valuable chemicals and fuels [3,4,5,6]. Among the reduction products, HCOOH plays a crucial role in various industries, including chemical production, liquid hydrogen storage, and fuel cells [7,8]. Hence, formate (formic acid) is considered a promising product with greater economic feasibility and industrial prospects among many CO_2_ reduction products [9]. However, electrochemical CO_2_RR still faces great challenges due to limitations in catalyst selection, such as low current density, high overpotential, poor selectivity, and stability [10,11].

Metals such as Sn, Bi, Pb, and In exhibit high selectivity for formic acid production during the electrochemical reduction of CO_2_ [12,13,14,15,16]. Due to its low cost and effective inhibition of hydrogen evolution reaction (HER), bismuth-based catalysts have become highlights of attention. Building dual or multi-component catalysts can effectively enhance CO_2_RR by exerting the synergistic effect of each component [17]. In particular, bimetallic catalysts can enhance the selectivity and activity of CO_2_RR by stabilizing the intermediates and inhibiting HER through synergistic effects [18]. Compared with single-metal catalysts, the advantage of bimetallics is that the binding energy of the reaction intermediates can be adjusted by changing the electronic structure and atomic arrangement [19]. Hence, the researchers focus on combining other metals with Bi to obtain bimetallic electrocatalysts with high selectivity, stability, and low overpotential, such as Bi-Sn, Bi-Cu, Bi-Ce, Bi-In, and Bi-Zn [20,21,22,23,24]. The bimetallic catalysts showed good catalytic activity and selectivity in the electrochemical CO_2_RR process for formic acid preparation. For instance, the strong binding energy between In and *OCHO compensates for the weak binding energy of Bi and *OCHO, which contributes to enhancing the selectivity of formic acid production [25].

Metal–organic frameworks (MOFs) have a unique framework structure, which is constructed from various organic ligands and inorganic metal clusters. The controllable properties and structure of MOFs make them ideal materials for catalyst optimization [26,27,28]. The porosity of MOFs promotes the transport of reactants to active sites [29]. The large specific surface area of MOFs also provides a large number of catalytic active sites, making them suitable for the preparation of electrocatalysts. The metal active sites in MOFs can exist in inorganic nodes or be embedded in the structure of the original MOF. Usually, the synthesized modified cations and different coordination centers provide different coordination environments. Their chemical environment exhibits excellent properties in coordination, which helps to gain a deeper understanding of how active sites affect their catalytic properties. In addition, the chemical environment can be further adjusted by selecting different types of metal centers [30]. Compared to monometallic MOFs, bimetallic MOFs can adjust the binding energies of the reaction intermediates by tuning the electronic structure and atomic arrangement [31,32]. However, there has been little research on combining the two into one entity due to the inherent low-current-density problems.

Herein, the metals Zn, In, Sn, and Bi were coordinated with the organic ligand 3-amino-1H-1,2,4-triazole-5-carboxylic acid (H_2_atzc) through a rapid microwave synthesis approach to construct bimetallic MOF materials (BiZn-MOF, BiSn-MOF, and BiIn-MOF). The bimetallic coordination centers in the MOF catalyst were regulated to optimize the catalytic performance of CO_2_RR. The optimized BiZn-MOF exhibited higher catalytic activity than those of Bi-MOF, BiSn-MOF, and BiIn-MOF. BiZn-MOF exhibited a higher selectivity for formate production with a faradic efficiency (FE = 92%) at a potential of −0.9 V (vs. RHE). The reaction intermediates were monitored by in situ Fourier transform infrared spectroscopy (FT-IR). The results indicated that the electrochemical conversion of CO_2_ to formate mainly follows the *OCHO pathway. The catalyst exhibited not only good electrocatalytic performance but also superior and reduced manufacturing costs compared to the pure metal catalyst (Sn catalyst) [33,34]. This work offers an effective way for the electroreduction of CO_2_ to formate and provides design ideas for constructing other efficient and economical catalysts.

## 2. Results and Discussion

### 2.1. Morphology and Structure of Bimetallic MOF

**Figure 1:**
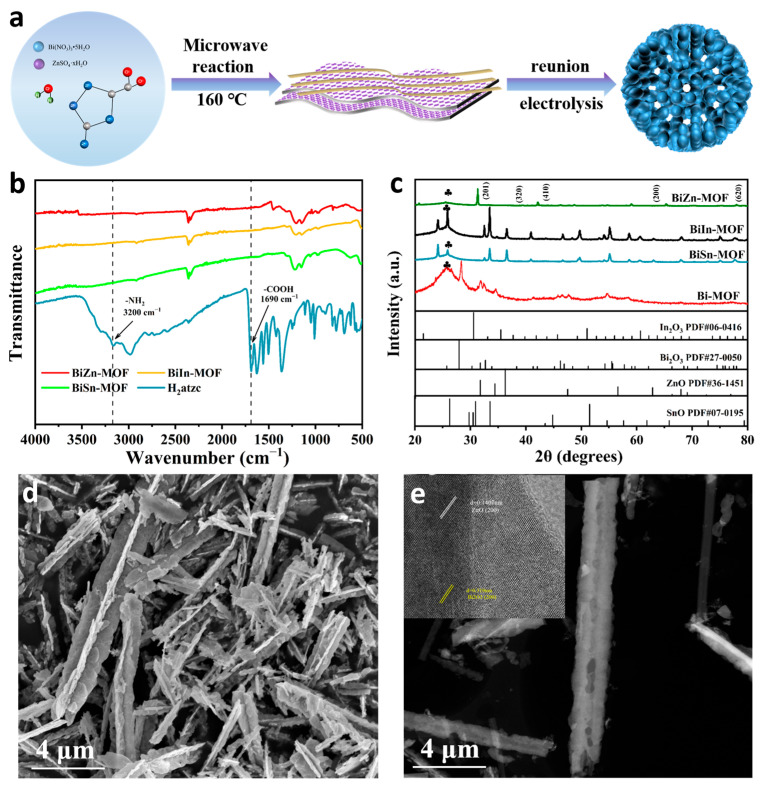
(**a**) Scheme illustration of the synthetic route for BiZn-MOF. (**b**) FT-IR spectra. (**c**) XRD patterns. The symbol ♣ indicates the characteristic peak of carbon paper. (**d**) SEM images of BiZn-MOF. (**e**) TEM and HRTEM images of BiZn-MOF.

Figure 1a shows the synthesis procedure of BiZn-MOF. The synthesis routes of BiIn-MOF and BiSn-MOF are similar to that of BiZn-MOF. By using 3-amino-1H-1,2,4-triazole-5-carboxylic acid hydrate (H_2_atzc) as an organic ligand, the catalysts BiZn-MOF, BiIn-MOF, and BiSn-MOF were successfully prepared through a rapid microwave approach. To verify the coordination environments of BiZn-MOF, BiIn-MOF, and BiSn-MOF catalysts, Fourier transform infrared spectroscopy (FT-IR) was performed, as shown in Figure 1b. The infrared absorption bands in the range of 3400 cm^−1^ to 3500 cm^−1^ for BiZn-MOF, BiIn-MOF, and BiSn-MOF catalysts can be attributed to O-H absorption bands in water molecules. H_2_atzc exhibits a stretching vibration at 1690 cm^−1^ due to C=O in the carboxyl group, and a plane stretching vibration mode at 3200 cm^−1^ due to N-H in the amino group. It is worth noting that the characteristic peaks at 1690 cm^−1^ and 3200 cm^−1^ did not appear in the BiZn-MOF, BiIn-MOF, and BiSn-MOF catalysts. Therefore, the carboxyl and amino functional groups of the organic ligand H_2_atzc successfully coordinated with the metal centers. Powder X-ray diffraction (XRD) patterns were conducted to further investigate the phase structure of BiZn-MOF, BiIn-MOF, BiSn-MOF, and Bi-MOF catalysts. Figure 1c shows that the diffraction peaks of all four catalysts match their respective characteristic peaks. The pattern of BiZn-MOF corresponded to the characteristic peaks of standard cards (PDF#27-0050) and (PDF#36-1451), respectively. The characteristic peaks at 27.95°, 42.05°, 48.44°, and 77.99° correspond to the (201), (320), (410), and (620) crystal faces of Bi_2_O_3_, respectively. The characteristic peaks at 31.76°, 34.42°, 47.53°, 56.60°, and 66.38° correspond to the (100), (002), (102), (110), and (200) crystal faces of ZnO, respectively. These results suggested that some oxides were formed on the MOF surface. A combination of XRD and FTIR indicated the successful preparation of BiZn-MOF, BiIn-MOF, and BiSn-MOF catalysts.

Figure S1a–c and Figure 1d showed the scanning electron microscope (SEM) images of the catalysts Bi-MOF, BiIn-MOF, BiSn-MOF and BiZn-MOF, respectively. The catalyst Bi-MOF has a spindle-like structure, but the bimetallic MOF showed a different morphology due to the incorporation of different metal ions Zn^2+^, Sn^4+^, and In^3+^. From SEM images, the catalyst BiZn-MOF presented a nanorod morphology, which were nano-assemblies formed by the accumulation of nanosheets and nanowires. The catalyst BiIn-MOF is an irregular nanosheet. The catalyst BiSn-MOF has a thick flaky nanoflower structure. The Brunner–Emmet–Teller (BET) technique was used to measure the specific surface area of the Bi-BTC catalyst, as shown in Figure S1d. There are a large number of pores on the surface of the nano-rod-like BiZn-MOF catalyst and the pore volume is 0.231 cm^3^/g. The catalyst has a specific surface area of 8.717 m^2^/g, which provides more active sites for the reaction interface. The unique structure and large specific surface area of BiZn-MOF provide a theoretical basis for excellent electrocatalytic performance in subsequent electrochemical CO_2_RR.

Transmission electron microscopy (TEM) was used to further determine the morphology and composition of the catalysts. Figure 1e reveals that the morphology of BiZn-MOF was nanorods. The top right diagram in Figure 1e shows obvious lattice streaks of ZnO and Bi_2_O_3_ in a high-resolution transmission electron microscope (HRTEM), with a lattice spacing of 0.141 nm and 0.319 nm corresponding to the (200) crystal face of ZnO and the (201) crystal face of Bi_2_O_3_, respectively. The scanning map of element distribution in Figure S2a–d confirmed that the morphology of BiZn-MOF is a nano-composite formed by the accumulation of nanosheets and nanowires. All elements are evenly distributed, which proves the successful preparation and synthesis of BiZn-MOF. Energy dispersive X-ray spectrum (EDX, Figure S2e) verifies the existence of Bi and Zn elements and uniform distribution on the catalyst BiZn-MOF. The atomic content of Bi and Zn was 33% and 0.03%, respectively.

**Figure 2:**
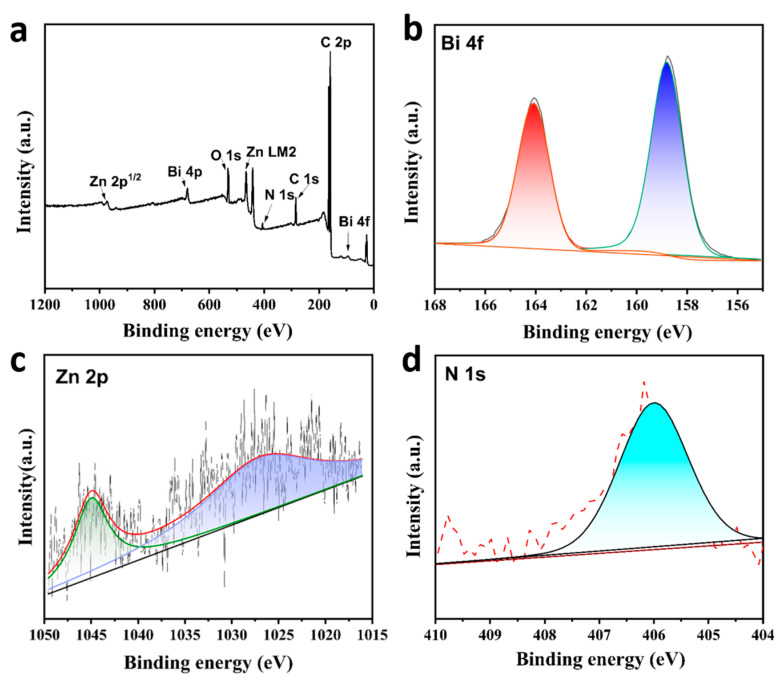
(**a**) XPS survey spectra of BiZn-MOF, (**b**) Bi 4f spectrum of BiZn-MOF, (**c**) Zn 2p spectrum of BiZn-MOF and (**d**) N 1s spectrum of BiZn-MOF.

The chemical composition and valence state of the BiZn-MOF catalyst were analyzed by XPS. The obtained XPS spectra were calibrated by aligning the position of peak C(sp2) in the C 1s spectrum with its reference value of 284.4 eV. Figure 2a shows the full XPS spectra of BiZn-MOF, which consists of elements Bi, Zn, C, O, and N. In Figure 2b, the sub-peaks at 165 eV and 159 eV in the Bi 4f spectrogram indicate the presence of metal ion Bi^3+^. The weak peaks at 1045 and 1028 eV in Figure 2c correspond to the Zn 2p in the catalyst. In Figure 2d, the N 1s spectrum at 406 eV demonstrated the existence of N-H bonding in the MOF catalyst.

### 2.2. Electrocatalytic Performance of CO_2_RR

**Figure 3:**
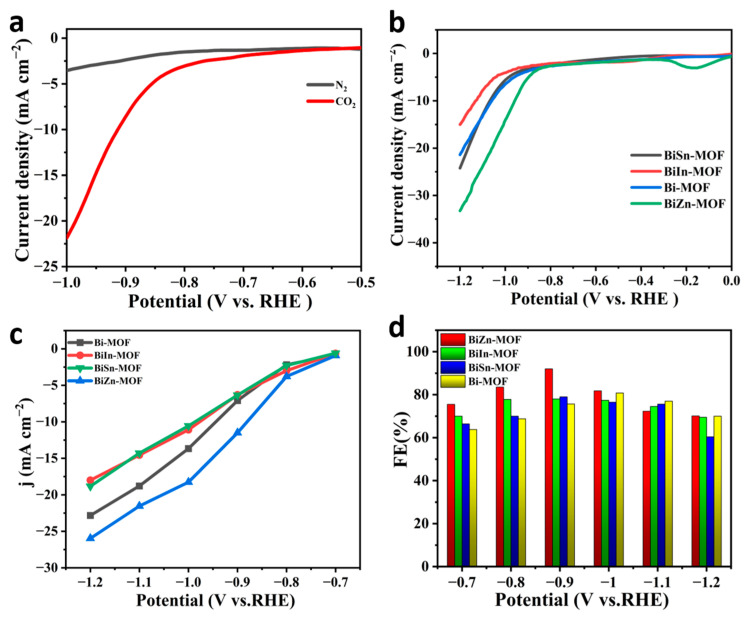
(**a**) Polarization curves of BiZn-MOF in saturated CO_2_ and N_2_ atmospheres, respectively; (**b**) Polarization curves of BiZn-MOF, BiIn-MOF, BiSn-MOF, and Bi-MOF catalysts in saturated CO_2_ electrolyte; (**c**) Local current densities; (**d**) Faraday efficiency of formate.

**Figure 4:**
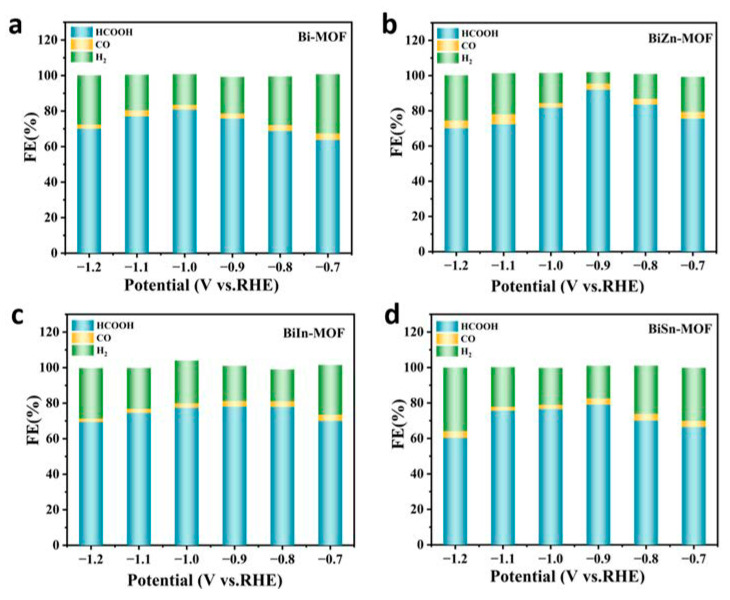
FEs of HCOO^−^, H_2_, and CO: (**a**) Bi-MOF, (**b**) BiZn-MOF, (**c**) BiIn-MOF, and (**d**) BiSn-MOF.

To evaluate the electrochemical properties of BiZn-MOF, BiIn-MOF, BiSn-MOF, and Bi-MOF catalysts, linear sweep voltammetry (LSV) curves were first tested using an H-type cell in an electrolyte saturated with CO_2_ or N_2_. As shown in Figure 3a, the current density of BiZn-MOF to CO_2_RR in a saturated CO_2_ electrolyte is obviously higher than that in a saturated N_2_ electrolyte. This result indicates that the catalyst BiZn-MOF exhibits good electrochemical CO_2_RR performance and can significantly suppress the generation of by-products (H_2_ and CO). The electrochemical CO_2_RR current density of BiZn-MOF is higher than that of BiIn-MOF, BiSn-MOF, and Bi-MOF, indicating better catalytic activity for BiZn-MOF compared to those for BiIn-MOF, BiSn-MOF, and Bi-MOF, as shown in Figure 3b. From Figure 3c, the catalyst BiZn-MOF has higher current density and corrected initial potential than the BiIn-MOF, BiSn-MOF, and Bi-MOF catalysts, indicating its superior electrocatalytic performance for CO_2_RR. Figure 3d illustrates the Faraday efficiency (FE_formate_) of formate products by four catalysts. The BiZn-MOF exhibits the best catalytic performance among two-component catalysts. At a potential of −0.9 V (vs. RHE), the FE_formate_ reached 92%, which was superior to Bi-MOF (with an FE_formate_ of 78%). This indicates that successful coordination of the dual metal center enhances formate selectivity, and BiZn-MOF exerts a bimetallic synergistic effect. Compared with the single component Bi-MOF, BiZn-MOF improves the selectivity of electrocatalytic CO_2_ and promotes the formation of formate. Figure 4a–d present a “volcano” diagram showing the selectivity of electrochemical CO_2_RR for various products (HCOOH, CO, and H_2_) prepared by four catalysts at different potentials. The peak values of BiZn-MOF, BiIn-MOF, BiSn-MOF, and Bi-MOF formate are 92%, 78%, 79.5%, and 82%, respectively. BiZn-MOF exhibits the highest selectivity for formate.

To further investigate the effect of the electrochemical active surface area (ECSA), cyclic voltammetry (CV) curves were obtained for four catalysts (Bi-MOF, BiZn-MOF, BiIn-MOF, and BiSn-MOF) at different sweep speeds (20–120 mV s^−1^), as shown in Figure S3a–d. The linear curve was plotted with sweep speed on the horizontal axis and current density difference on the vertical axis. The slope C_dl_ of the linear regression line reflects the magnitude of ECSA. Figure S3e shows that the C_dl_ (180 μF cm^−2^) of BiZn-MOF in the two-component catalyst is similar to that of Bi-MOF (150 μF cm^−2^). This result confirmed that the differences in CO_2_RR performance among the four catalysts mainly depend on optimizing the bimetallic centers, not the ECSA. Electrochemical impedance spectroscopy (EIS) was used to study electrode reaction kinetics at the interface between the electrode and electrolyte. From the EIS in Figure S4a, the semi-circle radius of BiZn-MOF in the two-component catalyst is the smallest (6 Ω), smaller than that of Bi-MOF (27 Ω), BiIn-MOF (9 Ω), and BiSn-MOF (8 Ω). This reflects that with the insertion of Zn^2+^, the charge transfer rate of BiZn-MOF is accelerated, thus boosting the catalytic activity to CO_2_RR. To better understand the reaction kinetics of BiZn-MOF, BiSn-MOF, and BiIn-MOF catalysts, Tafel slope analysis was performed. As shown in Figure S4b, the Tafel slopes of BiZn-MOF (106 mV dec^−1^) and BiIn-MOF (104 mV dec^−1^) are lower than that of BiSn-MOF (140 mV dec^−1^). These results indicate that the electron transfer rate of BiZn-MOF and BiIn-MOF catalysts is fast, which is conducive to the adsorption and desorption of *CO on their surfaces.

**Figure 5:**
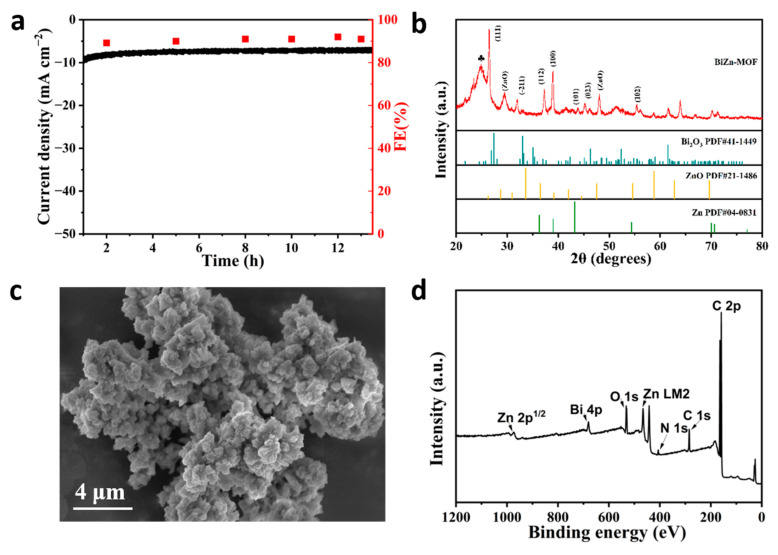
(**a**) Long-term stability of BiZn-MOF at −0.9 V (vs. RHE) potential and FE of HCOO^−^. Characterizations of BiZn-MOF catalyst after electrolysis for 10 h: (**b**) XRD pattern. The symbol ♣ indicates the characteristic peak of carbon paper. (**c**) SEM image of BiZn-MOF. (**d**) XPS spectra of BiZn-MOF.

Figure S5a–c display the constant potential electrolysis of CO_2_ at various potentials. The stable current density indicates that BiZn-MOF, BiIn-MOF, and BiSn-MOF catalysts exhibit good electrochemical stability in the CO_2_RR. By further exploring the stability of CO_2_RR materials, BiZn-MOF decomposes for about 13 h at a potential of −0.9 V (vs. RHE) in Figure 5a. The current density of BiZn-MOF remains stable at 13 mA cm^−2^, and the Faraday efficiency of producing formate products by electrochemical CO_2_RR is approximately 92%. These results indicate that BiZn-MOF exhibits good stability towards CO_2_RR.

XRD was used to further confirm the phase purity and composition of the BiZn-MOF catalyst after electrolysis, as shown in Figure 5b. It was observed that new characteristic diffraction peaks appeared after CO_2_ electrolysis. By comparing the standard cards (PDF#04-0831) and (PDF#41-1449), diffraction peaks at 36.29°, 38.99°, 43.23°, 54.33°, 70.66°, and 77.02° corresponded to crystal faces (002), (100), (101), (102), (110), and (004) of Zn, respectively. The angles of 26.92° and 32.48°corresponded to Bi_2_O_3_’s crystal faces of (111) and (−211). This is because some Zn^2+^ was reduced to Zn after the electrolysis of the catalyst BiZn-MOF. The SEM image in Figure 5c reveals that agglomeration occurred on the surface of BiZn-MOF after electrolysis, mainly due to structural changes caused by the partial reduction of Zn^2+^. Figure 5d shows survey XPS spectra, indicating that BiZn-MOF still contains elements Bi, Zn, C, N, and O after electrolysis. The XPS spectrum of Bi 4f in Figure S6a reveals the presence of metal ion Bi^3+^ at peaks of 165 eV and 159 eV, which was consistent with the results from the XRD pattern. In Figure S6b, the Zn 2p spectrum shows that the binding energy at 1021 eV corresponds to the diffraction peak of Zn (0), and the peak signal changed, mainly due to the low content of Zn in the BiZn-MOF catalyst and partial shedding (wt = 0.02%) after electrolysis. In Figure S6c, N 1s spectra show the same coordination pattern as that of the pre-electrolysis catalyst BiZn-MOF. In Figure S7a, the energy dispersive X-ray spectrometer (EDX) shows that Bi and Zn elements were evenly distributed on the catalyst BiZn-MOF after electrolysis, which verified the presence of Zn elements post-electrolysis. The content of Bi and Zn is 69.66% and 0.02%, respectively, indicating that some Zn may have been lost during the electrolysis process of BiZn-MOF, resulting in a decrease in its content. The element distribution mapping further confirmed that agglomeration occurred on the surface of the BiZn-MOF catalyst after electrolysis, while the elements C (blue), N (purple), O (yellow), Bi (red), and Zn (green) were uniformly distributed. 

**Figure 6:**
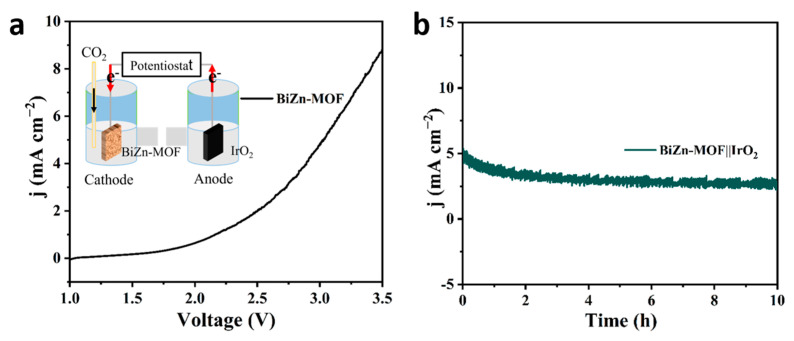
Performance of the CO_2_RR∥OER full cell for overall CO_2_ splitting: (**a**) LSV curves for the CO_2_RR∥OER full cell based on the BiZn-MOF∥IrO_2_ pair and (**b**) I-t curves at 3.0 V in CO_2_RR∥OER full cell.

Electrochemical CO_2_RR is a promising electrocatalytic technology, but due to the slow kinetics of oxygen evolution (OER) at the anode during electrolysis, a large amount of energy is needed. BiZn-MOF exhibited excellent electrocatalytic activity and selectivity in the process of electrocatalytic CO_2_ reduction. To confirm the practical application of the BiZn-MOF catalyst, a whole electrolytic cell was assembled by coupling the cathode material BiZn-MOF with the anode material IrO_2_. Figure 6a shows that a current density of 9 mA cm^−2^ can be achieved with a cell voltage of 3.5 V and 5 mA cm^−2^ at 3.0 V. After constant potential current–time curve (I-t) test on CO_2_RR‖OER (Figure 6b), it is found that BiZn-MOF‖IrO_2_ can maintain stability for up to 10 h at 3.0 V, indicating application prospects for BiZn-MOF‖IrO_2_, despite the expensive anode material IrO_2_, which has good stability and OER catalytic activity.

### 2.3. Catalytic Mechanism

**Figure 7:**
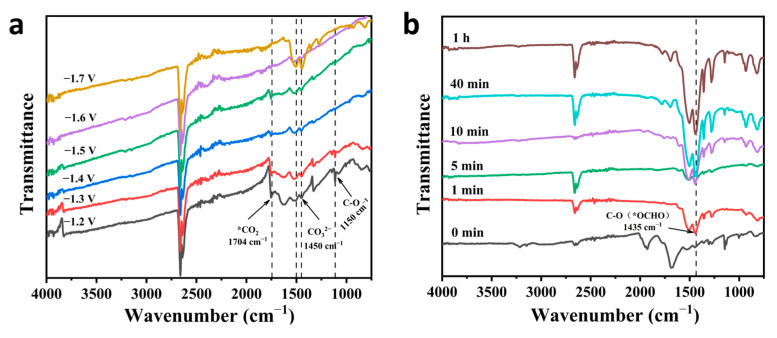
In situ FTIR of BiZn-MOF: (**a**) electrolysis at a potential of −1.2 V to −1.7 V for 1 h, and (**b**) at a potential of −0.9 V (vs. RHE) for different electrolysis times.

To better study the reaction pathway and mechanism of electrocatalytic CO_2_RR for formate formation by a catalyst, in situ Fourier transform infrared spectroscopy (FT-IR) was used to detect catalytic reaction intermediates. The FT-IR spectra can reflect the molecular structure, identify structural composition, and determine the presence of chemical groups [35], thus enabling the detection of reaction intermediates. In Figure 7a, FTIR of BiZn-MOF was tested in a CO_2_-saturated 0.5 M KHCO_3_ electrolyte with different potential windows (−1.2V to −1.7 V) for electrolysis. Distinct peaks of *CO_2_ intermediates are observed within the wavelength range of 1150 cm^−1^ and 1704 cm^−1^, which play a crucial role in electrocatalytic CO_2_ reduction to formate. Additionally, the characteristic absorption peak of CO_3_^2−^ is observed at 1450 cm^−1^, while the stretching vibration of the C-O bond in CO_3_^2−^ manifests at 1510 cm^−1^ [36]. The symmetrical tensile vibration of OCO in *OCHO intermediates is observed at 1435 cm^−1^. The vibration pattern of OCO was also observed during the adsorption of *OCHO intermediates and formate. The FTIR in Figure 7b displays a distinctive peak at 1435 cm^−1^. With the intensity gradually increasing as the electrolysis time extends, indicating an increase in the vibration mode of OCO. This result further clarifies the electrochemical CO_2_RR to formate followed the *OCHO reaction pathway.

When preparing formate (or formic acid) via electrochemical CO_2_RR, two reaction pathways exist [37]:

CO_2_ + H^+^ + e^−^ → *OCHO (or *COOH) + H^+^ + e^−^ → HCOOH

The final transformation of the reaction intermediate into either HCOOH or CO is affected by the pH of the electrolyte, which is a key factor in determining the product. Generally speaking, alkaline conditions are more conducive to forming *OCHO intermediates [38,39,40]. This study employs a 0.5 M KHCO_3_ solution (pH = 8.2) with an alkaline condition, so it is more conducive to *OCHO formation, thus obtaining e^−^ to generate formate (or formic acid). Simultaneously, *OCHO exhibits enhanced affinity towards the bimetallic active centers Bi and Zn in catalytic material BiZn-MOF, leading to the formation of Bi-O and Zn-O bonds that ensure surface stability [41]. Due to the strong electron-withdrawing ability of *COOH, it favors the formation of Bi-C or Zn-C bonds and C-C coupling [42]. However, in this study, only HCOOH and CO products were detected instead of C2 products, which further supports the proposed *OCHO reaction pathway.

## 3. Materials and Methods

### 3.1. Synthesis of Bimetallic MOF Catalysts

Firstly, ZnSO_4_·xH_2_O (69.41 mg) and Bi (NO_3_)_3_·5H_2_O (340 mg) were dissolved in deionized water (60 mL), and then mixed with H_2_atzc (89.7 mg). Subsequently, it was stirred at room temperature for 30 min and transferred to a microwave reactor at 160 °C for 1.5 h. Finally, the catalytic materials BiZn-MOF were centrifuged three times and then put into a drying oven at 60 °C to dry the samples. The other three catalysts (BiIn-MOF, BiSn-MOF, and Bi-MOF) were prepared with a similar procedure to BiZn-MOF by changing the metal sources.

### 3.2. Electrochemical Measurements

The carbon paper was ultrasonic in acetone and deionized water for 1 h. Then, 5 mg of BiZn-MOF (or BiIn-MOF, BiSn-MOF, and Bi-MOF) was put into 50 μL Nafion solution, 500 μL deionized water, and 450 μL ethanol and ultrasound to prepare the catalyst ink. Finally, a dried carbon paper (1 cm × 1 cm) with a load of 1 mg/cm^2^ catalyst was prepared by transferring appropriate ink drops onto the carbon paper.

All electrochemical tests were carried out at room temperature, using a type H cell for this work. The cathode chamber and the anode chamber were separated by a proton exchange membrane. A certain volume of 0.5 M KHCO_3_ electrolyte was measured in the cathode chamber and the anode chamber. In the preparation stage of the experiment, the inlet flow rate was 20 mL/min and CO_2_ gas was ventilated for 40 min until the electrolyte in the cathode chamber reached saturation. A three-electrode system was adopted, with platinum sheet as the counter electrode and Ag/AgCl (saturated KCl) as the reference electrode and the working electrode in the cathode chamber. After that, the three electrodes were kept at the same height. All electrochemical tests were performed on the electrochemical work station (Shanghai Chenhua, CHI 760D, China) where the pH of the CO_2_-saturated electrolyte was 6.8. All potentials in this study were measured with respect to Ag/AgCl reference electrodes. The calculation formula is as follows:

$$E(vs.RHE)=E\left(vs.Ag/AgCl\right)+0.222V+0.0591\times pH$$

### 3.3. Product Analysis

The gas phase product was directly detected by gas chromatography (GC7900, Tianmei, China) after coming out of the gas outlet of the H-type electrolytic cell. The carbon-containing gas products in the cathode chamber were analyzed by a methane reformer and a flame ionization detector (FID). A thermal detector (TCD) was used to detect CO_2_RR byproduct H_2_. Gas products (CO, H_2_) are detected when the current tends to be stable. The Faraday efficiency of gas products is calculated as follows:

$$FE(H_{2}/CO)=\frac{v\times t\times C\times N\times F}{60\times 24\times 1000\times Q}\times 100\%=\frac{v\times C\times 2\times 96485}{60\times 24000\times Q}\times 100\%$$

where *ν* represents the supplied CO_2_ gas flow rate (20 mL/min), *C* denotes the concentration of H_2_ (or CO) in the GC sample ring, *N* is the number of electrons transferred by H_2_ or CO molecules (*N* = 2), *F* stands for Faraday efficiency (96,485 *C*/mol), and *Q* represents the amount of electrolytic charge.

The liquid products after electrolysis were detected by ion chromatography (AS-DV, Thermo Scientific, New York City, NY, USA). Several quantities of formate products were collected from the cathode chamber in the H-type electrolytic cell and injected into the ion chromatography. The concentration (ppm) of formate in liquid phase products was determined by the integral area of the HCOO^−^ standard curve. Faraday’s formula is as follows:

$${FE}_{formate}(\%)=\frac{n_{formate}\times N\times F}{Q}\times 100\%=\frac{C_{formate}\times V\times 2\times 96,485}{I\times t}\times 100\%$$

where *n*_formate_ is the molar amount of formate in the cathode chamber, *N* represents the number of electrons required to generate HCOO^−^, *F* denotes the Faraday constant (96,485 C/mol), and *Q* indicates the amount of electron transfer charge (C). For each potential, the liquid phase product in the cathode chamber was collected 3–4 times to obtain an average value. The liquid phase product was then detected using ion chromatography. The concentration of the liquid phase product was calculated based on the peak area of a standard curve, allowing for determination of the Faraday efficiency of formate production.

## 4. Conclusions

In summary, bimetallic catalysts BiZn-MOF, BiSn-MOF, and BiIn-MOF were successfully synthesized using a rapid microwave method. The optimized catalyst BiZn-MOF exhibits good catalytic activity for electrochemical CO_2_ reduction to formate. At a potential of −0.9 V (vs. RHE), the formate Faradaic efficiency reaches 92%. The good catalytic performance of BiZn-MOF may be attributed to the Bi-Zn bimetallic coordination centers in the MOF, which can reduce the binding energies of the reaction intermediates by tuning the electronic structure and atomic arrangement. This study presents a rational and low-cost design concept for bimetallic catalysts in CO_2_RR research using a facile microwave method.

## Data Availability

All data in this study can be found in public databases and Supplementary Information, as described in the Material and Methods section (Section 3).

## Supplementary Materials

The following supporting information can be downloaded at https://www.mdpi.com/article/10.3390/ijms241813838/s1.
