# Supplementary material for: Regulation of Bimetallic Coordination Centers in MOF Catalyst for Electrochemical CO2 Reduction to Formate
Source: Int J Mol Sci. 2023 Sep 8;24(18):13838. doi: 10.3390/ijms241813838 (PMC10530794; doi:10.3390/ijms241813838)
Supplement: Supplementary file 1 [file ijms-24-13838-s001.zip › ijms-2592933-supplementary.pdf]

Supplementary information

Regulation of Bimetallic Coordination Centers in MOF Catalyst for Electrochemical CO_2 Reduction to Formate

Rui Yang †, Qun Huang †, Xuelan Sha, Beibei Gao and Juan Peng *

State Key Laboratory of High-Efficiency Utilization of Coal and Green Chemical Engineering, College of Chemistry and Chemical Engineering, Ningxia University, Yinchuan 750021, China; 18709609803@163.com (R.Y.); 18709506169@163.com (Q.H.); shaxuelan2023@163.com (X.S.); gbb214428@163.com (B.G.).
* Correspondence: pengjuan@nxu.edu.cn
† These authors contributed equally to this work.

Chemicals and Reagents

The following reagents were purchased and used in this work without further purification: bismuth nitrate pentahydrate ($Bi(NO_3)_3 \cdot 5H_2O$, purity≥99%) was purchased from Tianjin Damao Chemical Reagent Factory. The organic reagent 3-amino-1H-1,2,4-triazole-5-carboxylic acid (H_2atzc, purity ≥95%) was obtained from Sinopharm Chemical Reagent Co., Ltd. Indium trichloride tetrahydrate ($InCl_3 \cdot 4H_2O$, purity≥99.99%), tin tetrachloride pentahydrate ($SnCl_4 \cdot 5H_2O$, purity ≥99%) and hydrated zinc sulfate ($ZnSO_4 \cdot xH_2O$, purity>95%) were purchased from Shanghai Macklin Biochemical Co., Ltd. Nafion solution (5.0wt %) was obtained from Shanghai Aladdin Biochemical Technology Co., Ltd. Potassium bicarbonate ($KHCO_3$, purity≥99.5%) was purchased from Sinopharm Chemical Reagent Co., Ltd. Deionized water (18.24 MΩ cm^{-1}) was produced by the laboratory special ultra-pure water machine.

Characterization and measurement

The transmission electron microscope (TEM) images were captured using a Japanese JEOL JEM-F200 microscope operating at 100 kV. Multiple high-resolution TEM images were obtained using a JED-2300T microscope with an acceleration voltage of 200 kV. High-angle toroidal dark-field scanning transmission electron microscopy (HAADF-STEM) images were acquired using FEITEM at 200 kV. Kα ray diffraction (XRD) characterization was performed on a Rigaku 9 kW XRD diffractometer. Kα ray photoelectron spectroscopy (XPS) analysis was conducted using the Thermo Scientific K-Alpha instrument, and the corresponding binding energies of Bi 4f and O 1s peaks were calibrated. The nitrogen adsorption-desorption isothermal curve was obtained at 100°C using Tristar II 3020. The amount of each sample used in the test was 72 mg. Two cycles of gas desorption were performed before the nitrogen adsorption experiment. The electrodes were characterized directly prior to the electrocatalytic reaction occurring. After the electrocatalytic reaction, the electrode was cleaned with deionized water to remove surface electrolyte and then characterized after drying.

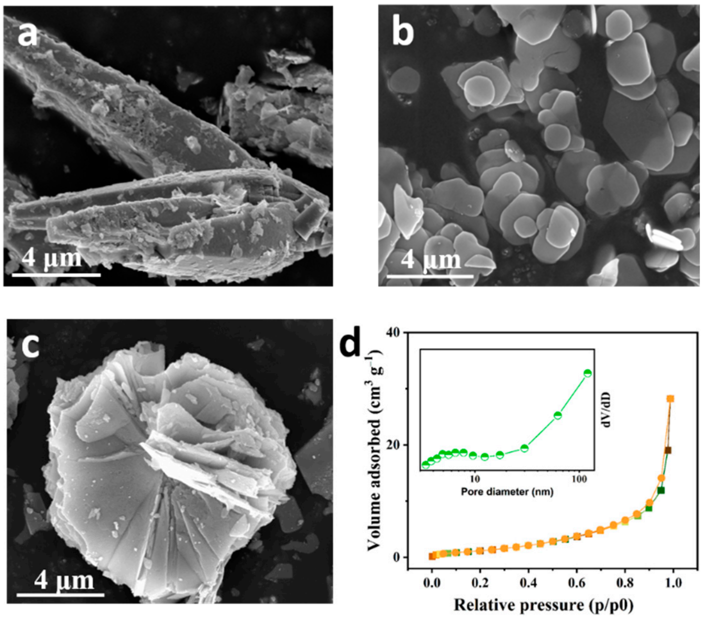

Figure S1. SEM images of (a) Bi-MOF, (b) BiIn-MOF and (c) BiSn-MOF. (d) Nitrogen adsorption-desorption isotherm curves of BiZn-MOF samples.

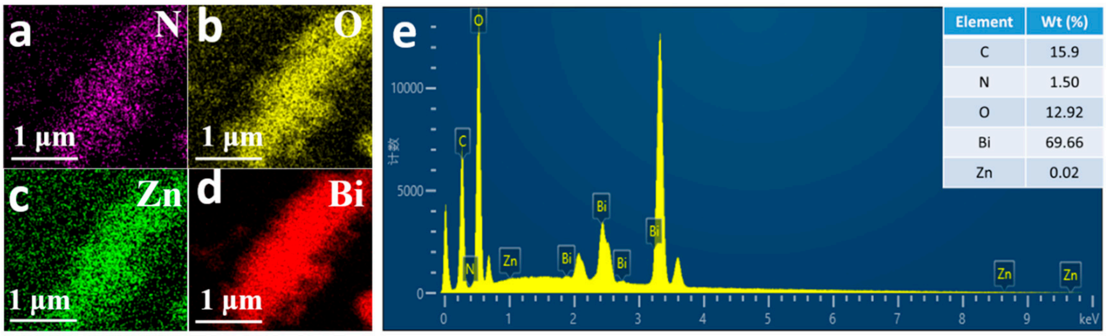

Figure S2. (a-d) Elemental mapping image of BiZn-MOF (purple, yellow, green and red represent N, O, Zn, Bi, respectively). (e) EDX spectra of BiZn-MOF catalyst.

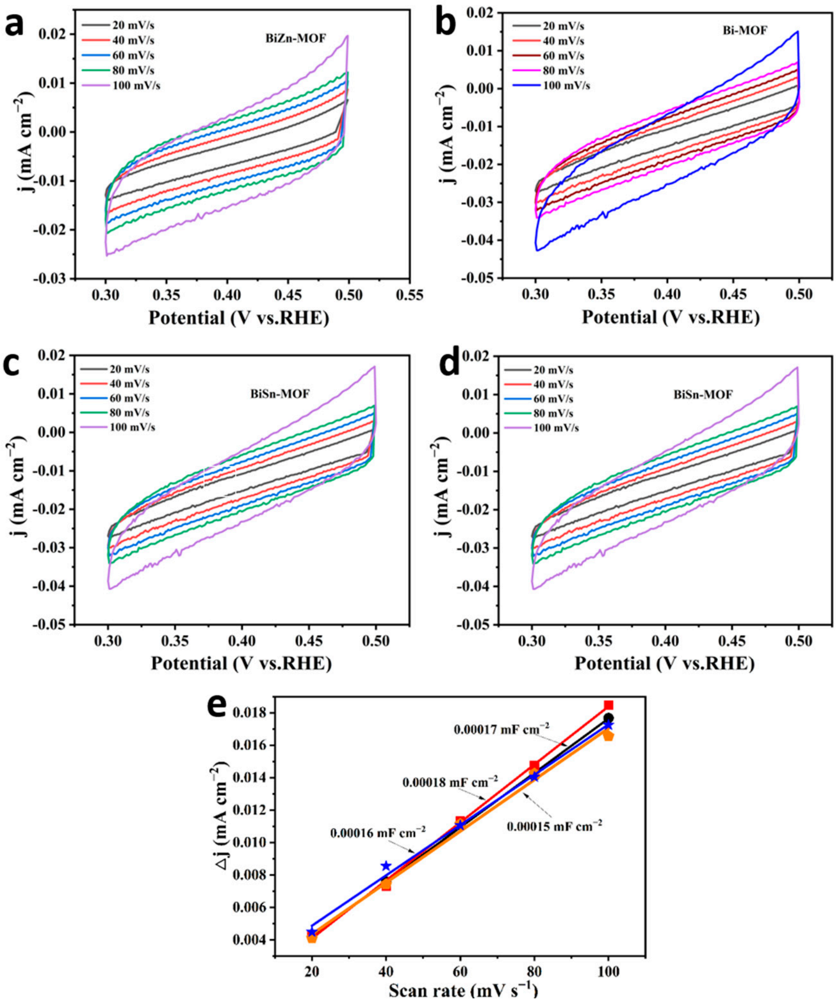

Figure S3. CV curves of (a) BiZn-MOF, (b) Bi-MOF, (c) BiIn-MOF, and (d) BiSn-MOF in the potential range from 0.27 V to 0.53 V at different scanning rates (20-100 mV s^{-1}). (e) Linear fitting graph of current density difference △j with sweep speed (red, black, blue and orange represent BiZn-MOF, BiIn-MOF, BiSn-MOF and Bi-MOF, respectively).

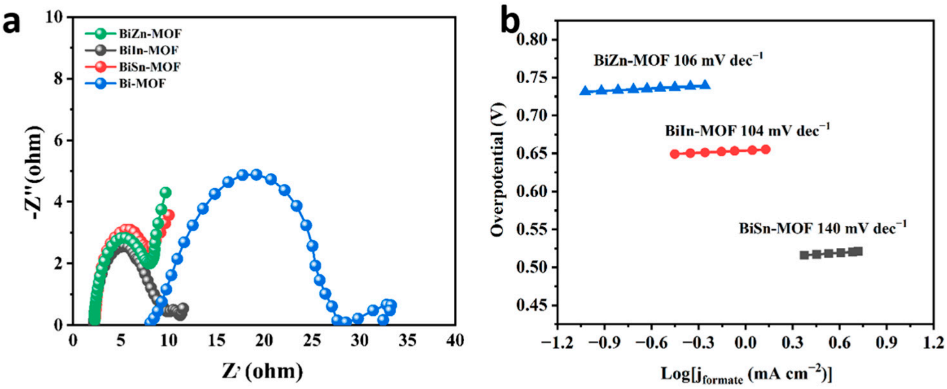

Figure S4. (a) Electrochemical impedance spectroscopy of BiZn-MOF, BiIn-MOF, BiSn-MOF and Bi-MOF. (b) Tafel slope of BiZn-MOF, BiIn-MOF and BiSn-MOF.

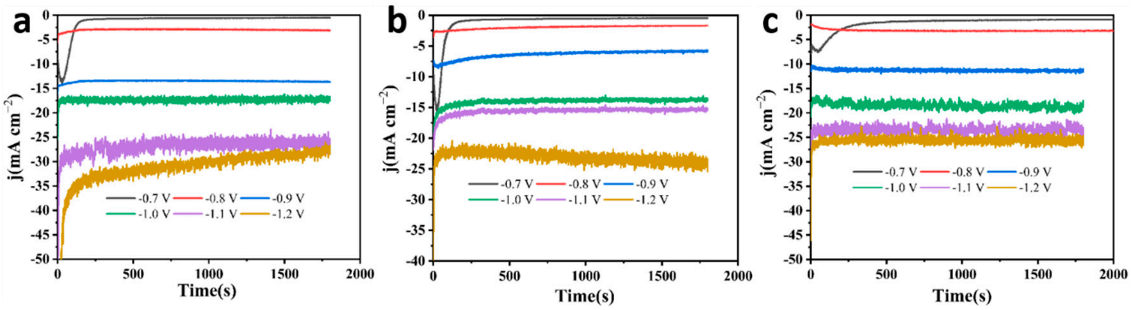

Figure S5. Chronoamperometric responses of (a) BiZn-MOF, (b) BiIn-MOF, (c) BiSn-MOF at different working potentials as indicated for 1 h.

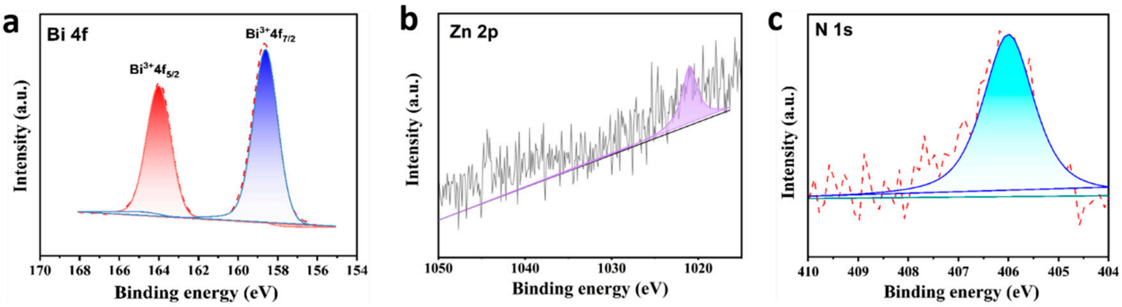

Figure S6. (a) Bi 4f XPS spectrum. (b) Zn 2p XPS spectrum. (c) N 1s XPS spectrum.

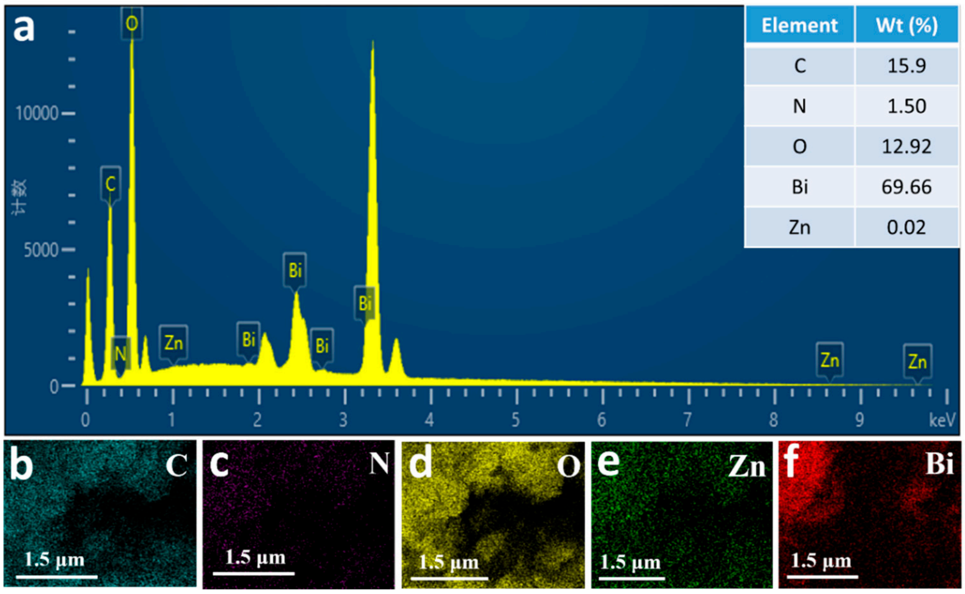

Figure S7. Characterization of BiZn-MOF after 13 hours of electrolysis: (a) EDX image and (b-f) elemental mapping image (blue, purple, yellow, green and red represent C, N, O, Zn and Bi, respectively).
